# Metabolic Mechanisms Connecting Alzheimer’s and Parkinson’s Diseases: Potential Avenues for Novel Therapeutic Approaches

**DOI:** 10.3389/fmolb.2022.929328

**Published:** 2022-06-16

**Authors:** Jerry R. Colca, Brian N. Finck

**Affiliations:** ^1^ Metabolic Solutions Development Company, Western Michigan University, Kalamazoo, MI, United States; ^2^ Washington University School of Medicine, St. Louis, MO, United States

**Keywords:** Parkinsion’s disease, Alzhaimer’s disease (AD), mitochondria, autophagy, inflammation

## Abstract

Alzheimer’s (AD) and Parkinson’s Diseases (PD) are common neurodegenerative disorders growing in incidence and prevalence and for which there are no disease-modifying treatments. While there are considerable complexities in the presentations of these diseases, the histological pictures of these pathologies, as well as several rare genetic predispositions for each, point to the involvement of maladaptive protein processing and inflammation. Importantly, the common presentations of AD and PD are connected to aging and to dysmetabolism, including common co-diagnosis of metabolic syndrome or diabetes. Examination of anti-diabetic therapies in preclinical models and in some observational clinical studies have suggested effectiveness of the first generation insulin sensitizer pioglitazone in both AD and PD. Recently, the mitochondrial pyruvate carrier (MPC) was shown to be a previously unrecognized target of pioglitazone. New insulin sensitizers are in development that can be dosed to full engagement of this previously unappreciated mitochondrial target. Here we review molecular mechanisms that connect modification of pyruvate metabolism with known liabilities of AD and PD. The mechanisms involve modification of autophagy, inflammation, and cell differentiation in various cell types including neurons, glia, macrophages, and endothelium. These observations have implications for the understanding of the general pathology of neurodegeneration and suggest general therapeutic approaches to disease modification.

## Overview

Drug discovery efforts generally focus on narrowly defined syndromes in search of precision solutions. However, effective therapeutics for neurodegenerative diseases remain a significantly unmet medical need likely because of the broad and complex presentations and the limited heritability of these progressive diseases. Alzheimer’s disease (AD) is the most common cause of age-related dementia and manifests in a variety of presentations and symptoms (Scheltens et al., 2021). Work in twins suggests that AD, as currently diagnosed, may be 60–80% heritable and over 40 risk-associated genetic variations have been identified. Some these mutations seem to fit with the histological presentation of disease, and indeed these insights have facilitated the generation of animal models that can recapitulate aspects of AD pathology. Parkinson’s disease (PD), the most common form of neurodegeneration associated with movement disorders, is also partially heritable with some mutations that can also produce symptoms in animal models (Simon et al., 2020). In this case, motor symptoms can also be produced by specific toxins, which provides relevant information as to pathophysiology, particularly with respect to the loss of function in the dopamine system.

Unfortunately, while advances in understanding the genetics of some rare subsets of AD and PD have identified rational therapeutic targets and some animal models have been created, effective disease modifying agents in clinical trials have not been forthcoming. Efforts are complicated by the fact that individual mutations in the spectrum of AD and PD, even when they reflect a component of the pathological phenotype, describe at best a very small percentage of people with these diseases. Disease modifying therapeutics have remained elusive and these neurodegenerative diseases remain a significant affliction of aging and a major unmet need in global health care.

Herein, we discuss an alternate approach to treating neurodegenerative diseases by correcting the upstream metabolic drivers that are in common across neurodegenerative diseases. We summarize data generated from the growing descriptions of the pathophysiology of AD and PD and the emerging understanding of the molecular action of compounds known as insulin sensitizers. The confluence of these data suggests an approach to slowing disease progression by reprogramming metabolism. Metabolism impacts myriad cellular and molecular processes that modulate known pathophysiological mechanisms including autophagy, inflammation, and cell differentiation in multiple cell types. Thus, a metabolic approach would not only impact multiple molecular pathways in parallel but would also have the potential to positively impact both AD and PD.

### Disease Incidence, Presentation, and Implications From Genetic Studies

Alzheimer’s disease (AD) is a common disease associated with aging that involves progressive loss of neurons and deposition of plaques composed of the Aβ protein. The prevalence of AD is continuing to increase, and it is currently the 5^th^ leading cause of death in the US for people over the age of 65. Critically, AD has an oversized impact on public health because of the level of care required as the disease progresses because patients may require intensive and round the clock care for many years. While there are clear differences in a clinical diagnosis of the various forms of AD that differ from aging p*er se*, age has a significant impact on disease progression regardless of the specifics of clinical diagnosis (Toepper, 2017; Guerrero et al., 2021; Scheltens et al., 2021). The physical presentation of AD is generally recognized as the increased presence of amyloid plaques and hyperphosphorylated tau which can be detected on autopsy and, more recently, by imaging techniques (Dani et al., 2016). Rare mutations in the amyloid pathway may predispose to early development of AD and insights from genetic data have led to the development of animal models with defects in amyloid metabolism that can mimic some of the AD pathology (Zhang et al., 2020). As a consequence of these insights, many of the drug discovery efforts directed at AD have targeted mechanisms that might reverse these histological manifestations (Jouanne et al., 2017; Lozupone et al., 2020; Tolar et al., 2020; Yu et al., 2021). While these plaques are the key signature of AD, there are many abnormalities in brain structure, function, and metabolism that have been discovered over the years.

PD is neurodegenerative disease with characteristic motor dysfunction. However, the clinical presentation often includes many non-motor defects ranging from constipation to neuropsychiatric issues (Simon et al., 2020; Vijiaratnam et al., 2021). The physical presentation includes loss of function of the dopamine producing neurons in the midbrain and can include cytosolic aggregations of misfolded protein that may include α-synuclein. Environmental toxins can trigger the loss of these motor neurons, but there are also rare mutations, including those coding for α-synuclein and proteins related to mitochondrial homeostasis, that predispose to the development of PD. In general, all the known risk-associated factors impinge on mitochondrial function, oxidative stress, impaired autophagy and neuroinflammation (Vijiaratnam et al., 2021). Like AD, the incidence of PD increases with age (Trist et al., 2019; Vijiaratnam et al., 2021). Thus, although the affected anatomical regions and the symptoms of these divergent neurodegenerative diseases are very different, there are similar factors involved in their pathological progression. As outlined below, these considerations point to a critical role for mitochondrial metabolism and frame an approach to interventions.

A summary of common factors that influence disease progression in both AD and PD is presented in Table 1. Of these commonalities, the relationship between metabolism and these neurodegenerative diseases is particularly interesting. There is a confluence among the associations of aging, metabolic syndrome and type 2 diabetes in incidence and progression of both AD and PD. Furthermore, a class of agents developed to treat the insulin resistance of type 2 diabetes has demonstrated positive clinical effects and, to the point of the current discussion, addressable molecular connections of the pathophysiology to metabolism.

**TABLE 1:** Common factors in AD and PD.

	AD	PD
Age	yes	yes
Mitochondrial function	yes	yes
Protein aggregates	amyloid	α-synuclein
Inflammation	yes	yes
Genetic/autophagy	yes	yes
Toxin-induced	no	yes
Metabolic/T2D	yes	yes

### Impact of Dysmetabolism

Although the details of molecular mechanisms remain the subject of much debate and investigation, metabolic dysfunction or dysmetabolism is likely both a cause and consequence of insulin resistance and type 2 diabetes mellitus (e.g., see (Czech, 2017; Roden and Shulman, 2019)). As discussed below, the spectrum of metabolic disturbance that includes type 2 diabetes is also strongly associated with AD and PD. While “normal” aging and metabolic dysfunction do not share all of the common neurodegenerative features of AD and PD, there are common underlying mechanisms that may contribute to disease progression (Tumminia et al., 2018; Frazier et al., 2019).

Abnormalities in brain insulin sensitivity are an early sign of AD. Indeed, many have described AD as “Type III diabetes” (de la Monte, 2014) due to many similarities in the metabolic abnormalities that are observed. There are a number of facets to metabolic dysfunction that impinge on progression of AD including the observation that glucose metabolism is impaired in affected brain regions in advance of cognitive decline. There is also a growing understanding of the importance of insulin and IGF-1 signaling in neural tissues (de la Monte, 2014; Arnold et al., 2018; Xue et al., 2019; Potenza et al., 2021). Indeed, blocking insulin signaling in a neuron-specific manner is sufficient to induce signs of Alzheimer’s in mice (Soto et al., 2019). An extensive literature is building in this respect, but here we will focus on the aspect of insulin resistance and mitochondrial metabolism that connects to the first therapeutic agents that were developed to address insulin resistance.

The association between type 2 diabetes and PD has also recently been reviewed (Aviles-Olmos et al., 2013; Cheong et al., 2020). Observational studies show an increase in incidence and severity of Parkinson’s symptoms in patients who have been co-diagnosed with diabetes. Moreover, patients with PD have been found to exhibit insulin resistance and impaired glucose tolerance, two manifestations of dysmetabolism coincident with diabetes. This has led to some small clinical trials using incretin agonist antidiabetic agents that have shown positive results (Athauda et al., 2017) suggesting the treatment of the underlying hyperglycemia could be useful. Observational clinical studies have shown a reduction in the incidence of PD in patients with diabetes who were treated the insulin sensitizing agents (Brauer et al., 2015; Hussain et al., 2020). Further scrutiny of these results is of particular interest because of the growing knowledge of the mitochondrial mechanism of action of these agents as discussed below.

Mitochondrial function is a key common feature of metabolism that connects AD, PD, and aspects of the metabolic syndrome including diabetes (Pinti et al., 2019; Wang et al., 2020a; Monzio Compagnoni et al., 2020; Weidling and Swerdlow, 2020; Borsche et al., 2021; Potenza et al., 2021). Mutations in the PINK1 and PRKN genes, which encode proteins that play important roles in regulating mitochondrial quality control, dynamics, and turnover, were among the first genes linked to development of PD (Malpartida et al., 2021). Because of this linkage to mitophagy, the autophagic processes that dispose of dysfunctional mitochondria, there has been a great deal of interest in PD therapeutics that stimulate autophagy or modulate mitochondrial function. Similarly, in AD, a number of defects in mitochondrial energetics, increased oxidative stress, fission/fusion, and turnover have been noted (Wang et al., 2020b). A number of potential triggers for mitochondrial abnormalities have been identified, but existing data suggest that mitochondrial dysfunction plays a causative role in the development and progression of AD. Therefore, targeting mitochondrial metabolism to treat AD has also garnered a great deal of attention.

Mitochondrial dysfunction initiates intracellular signaling cascades that can impair many processes known to be important in the progression of the disease including autophagy, inflammation, and cellular responses to growth factors. Obviously, mitochondrial dysfunction has implications for many cell types in all tissues (Pinti et al., 2019). It is important to note that this connection could be either because of a common underlying pathology, for example as occurs with aging, or because the metabolic dysfunction worsens other underlying etiologies. For example, buildup of misfolded proteins enabled by either a genetic predisposition and/or environmentally-induced loss of function can themselves precipitate exacerbation of the pathology creating a vicious cycle (Colca and Feinstein, 2012; Torres et al., 2021). Therefore, therapeutics that correct abnormalities in mitochondrial metabolism and elicit beneficial effects on processes like autophagy and neuroinflammation may be ideal candidates for treating neurodegenerative disease.

A review of the clinical studies of anti-diabetic drugs on the incidence of dementia in people with diabetes demonstrated a unique efficacy of pioglitazone (McMillan et al., 2018). Of particular relevance to this review, which seeks to connect the molecular mechanisms involved, a number of clinical observations and preclinical studies in neurodegenerative disease have also shown some intriguing results with pioglitazone. A brief background of pioglitazone is presented below in order to frame the discussion that follows.

### Insulin Sensitizers and Preclinical and Clinical Studies With Pioglitazone

In the 1990s, three drugs that improved insulin sensitivity (troglitazone, rosiglitazone, and pioglitazone) were approved for the treatment of type 2 diabetes. These drugs all share a common chemical structure; a thiazolidinedione (TZD) ring. As we have previously reviewed, thiazolidinediones were selected and developed in the mid 1980s based on their insulin sensitizing effects before there was a molecular hypothesis of their mechanism of action (Colca, 2006; Colca and Kletzien, 2006). Years after these compounds were selected and entered into clinical development, they were found to be direct activators of the transcription factor PPARγ and subsequently, many drug discovery programs were focused on finding additional and more potent PPARγ activators (reviewed in (Soccio et al., 2014)). However, it is now recognized that PPARγ activation is associated with the side effects that have plagued this class of drugs. Troglitazone, the first compound from this class to gain registration for treatment of type 2 diabetes, was removed the market due to compound-specific idiopathic hepatoxicity. Rosiglitazone was initially removed and then limited in use because of potential increases in cardiovascular disease and heart failure. Pioglitazone remains the only marketed therapeutic from this class that is still utilized clinically to any significant extent (Nissen and Wolski, 2007; Rizos et al., 2016; DeFronzo et al., 2019; Le et al., 2020). Even so, and although the pharmacology of pioglitazone has broader implications including prevention of cardiovascular and metabolic liver disease (DeFronzo et al., 2019; Le et al., 2020), it is used only as a second or third line anti-diabetic agent based on tolerability issues related to its direct activation of PPARγ, which results in fluid retention and the potential for bone loss. Interestingly, pioglitazone is 10-fold less potent PPARγ agonist compared to rosiglitazone and is as effective as an insulin sensitizer, but with a greater safety margin. This experience had suggested that other molecular targets may mediate some of its beneficial pharmacology (Colca and Kletzien, 2006).

Pertinent to this discussion, there have been numerous preclinical studies with TZDs showing multiple beneficial effects in models of neurodegeneration. In alignment with the earlier discussion on the importance of mitochondria in these pathologies, many of these studies have suggested that the actions of these agents on the mitochondria might be a key factor in the pharmacology (reviewed in 34). A more recent intriguing observational clinical study conducted in Germany demonstrated a 47% reduction in the incidence of dementia in people with diabetes who had been treated long-term with pioglitazone. In fact, for subjects treated more than 2 years, their risk of developing dementia was even lower than subjects who had not developed diabetes (Heneka et al., 2015). An excellent summary of all the preclinical and clinical results with TZDs has recently been published (Saunders et al., 2021). Although there have been many positive studies conducted and published with pioglitazone, a prospective study with very low dose pioglitazone was stopped for futility (Burns et al., 2021). The very low dose (0.8 mg) was used to avoid side effects that can be seen with the more commonly given clinical doses (30 and 45 mg). It is likely that this study failed because of the low exposures achieved, since another review of multiple clinical studies with pioglitazone points out that positive effects of pioglitazone on dementia are both dose- and time-dependent (Chou et al., 2017). The increased exposure required for clinical efficacy may be due to the requirement to engage other molecular targets such as the mitochondrial target for the TZDs.

### The Discovery of the Mitochondrial Target of TZDs and Implications to Mitochondrial Mechanisms

Although the general dogma is that pioglitazone acts via direct transcriptional regulation of the PPARγ nuclear receptor, as discussed earlier, pioglitazone was selected for development based on functional screens without regard or knowledge of mechanism. Subsequently, binding assays using subcellular fractions from both liver and brain demonstrated that tritiated pioglitazone binds primarily to mitochondrial membranes (Colca et al., 2004). Subsequent work using a pioglitazone-based, ^125^I-labeled photoaffinity crosslinker and unbiased proteomics revealed that the mitochondrial target of the TZDs was the mitochondrial pyruvate carrier (MPC) (Colca et al., 2013a; Colca et al., 2014). Studies conducted in multiple cell types confirmed that insulin-sensitizing TZDs that had been developed for treatment of type 2 diabetes acted as inhibitors of MPC activity by directly binding to the MPC complex (Divakaruni et al., 2013; McCommis et al., 2015; McCommis and Finck, 2015). The effects of pioglitazone on pyruvate metabolism may also be mediated via induction of pyruvate dehydrogenase kinase, which inhibits pyruvate oxidation by pyruvate dehydrogenase, and remodeling of mitochondrial lipids to alter mitochondrial function (Shannon et al., 2021). Thus, not only the entry of pyruvate but the modification of its downstream metabolism may be impacted by TZD use.

Two inner mitochondrial membrane proteins, MPC1 and MPC2, constitute the MPC, which is the obligate entry point of pyruvate into the mitochondrion (Bricker et al., 2012; Herzig et al., 2012; Sharma et al., 20192019). Since the primary routes of pyruvate synthesis are glycolysis or by lactate dehydrogenase, which are cytosolic processes, and the enzymes that metabolize pyruvate (pyruvate dehydrogenase and pyruvate carboxylase) are exclusively localized in the mitochondrial matrix, the MPC catalyzes an important regulatory step in intermediary metabolism. While it may seem counterintuitive that suppressing pyruvate metabolism is beneficial for treating metabolic disease, genetic deletion of the MPC in many tissues is well tolerated and protects mice from obesity and/or diabetes. For example, liver specific MPC deletion attenuated the flux of pyruvate into the gluconeogenic pathway to suppress liver glucose production in obese mice (McCommis et al., 2015). Deletion of the MPC in skeletal muscle protected mice from obesity and improved their metabolic profile in association with enhanced fatty acid oxidation (Sharma et al., 2019). Indeed, slowing the mitochondrial entry of pyruvate through the MPC results in enhanced use of other substrates including fatty acids and amino acids (McCommis et al., 2015; Divakaruni et al., 2017; Buchanan and Taylor, 2020), which may mediate some of the beneficial effects of MPC inhibition. Indeed, in addition to neurodegenerative diseases, identification of novel inhibitors of the MPC has garnered attention as a potential treatment of a variety of diseases including type 2 diabetes, NASH, cancer, and even hair loss (McCommis et al., 2017; Liu et al., 2021).

It is likely that MPC inhibition modulates the activity of various energy-sensing signaling cascades such as AMP-activated protein kinase (AMPK), NAD + -sensing deacetylases, or the mechanistic target of rapamycin (mTOR) kinase. Since these signaling pathways are known to regulate insulin sensitivity, inflammation, and autophagy, which are cellular processes known to be involved in the response to neurodegenerative stimuli, inhibition of the MPC may mediate beneficial effects by modulating the activity of these energy-sensing signaling cascades.

Alternatively, or in addition, other work has demonstrated a beneficial effect of MPC inhibition on neuronal death in response to excitatory amino acids. Divakaruni and colleagues showed that MPC inhibitors protected neurons at least in part by stimulating the metabolism of glutamate and other amino acids as an adaptive response to diminished flux through the MPC (Divakaruni et al., 2017). Since excitotoxic neuronal death has been implicated in the pathogenesis of neurodegenerative disease, the effects of MPC inhibition on the accumulation of excitotoxic amino acids could play a role in the therapeutic pharmacology.

Our understanding of the specific role of that the MPC plays in regulating metabolism in various cell types is now growing based on a variety of cell type-specific knockouts (Gray et al., 2015; McCommis et al., 2016; McCommis et al., 2017). The ability of insulin-sensitizing TZDs to impact mitochondrial pyruvate metabolism likely explains the pleotropic pharmacology that has been observed over the years since their original discovery (Colca, 2015). This pharmacology may include maintaining the functionality of adipocytes, thus uncoupling of body weight from insulin resistance (Colca and Scherer, 2022). The broad potential of MPC activity modulation with respect to neurodegeneration has recently been reviewed (Tang, 2019; Zangari et al., 2020) and will be discussed in greater detail below.

As it became evident that direct activation of PPARγ could be avoided while maintaining the interaction with the mitochondrial target, a medicinal chemistry program was directed *against* the direct activation of PPARγ (Chen et al., 2012; Tanis et al., 2018). This has led to development of new TZDs for multiple indications (Colca et al., 2013b). More recently screening efforts are demonstrating the possibility of finding new molecular scaffolds that can interact with and attenuate the flux of pyruvate through the MPC (Hegazy et al., 2022). It is not yet clear whether all of these new scaffolds will interact with the molecular target in a way that produces the same pleotropic pharmacology as the TZDs like pioglitazone (Colca, 2015).

### Studies With New TZDs

MSDC-0160 is an isomer of one the pioglitazone metabolites, which is reduced to a stereoisomer of another minor pioglitazone metabolite in both preclinical models and in man (Colca et al., 2013b). MSDC-0160 and its primary hydroxy metabolite have limited ability to bind to and directly activate PPARγ while maintaining the antidiabetic pharmacology of pioglitazone (Chen et al., 2012; Colca et al., 2013b; Tanis et al., 2018). The active metabolite of MSDC-0160 has also been shown to readily cross the blood-brain-barrier in rodent models (Peelaerts et al., 2020). The potential utility of this approach is supported by clinical efficacy and safety as discussed below.

MSDC-0160 has been studied in a Phase 2 dose-ranging study in subjects with diabetes where it demonstrated the same anti-diabetic activities as pioglitazone without evidence of dose-tolerability issues (Colca et al., 2013c). A 3-months Phase 2a clinical study of people with mild to moderate AD without diabetes evaluated the potential of MSDC-0160 treatment to maintain ^18^F-2-FDG PET uptake in the lateral temporal cortex, medial temporal cortex and anterior cingulate-medial frontal cortex, areas known to decline in parallel with measures of cognition. When these regions were compared to the cerebellum, the decline in the placebo group (N = 13) was prevented in the MSDC-0160-treated patients (N = 16). These results are supportive of a neuroprotective effect. There were also several other brain regions, typical of amyloid deposits, that demonstrated a treatment-induced *decrease* in the 18F-2-FDG PET uptake. This would be expected of an anti-inflammatory action, which the authors suggest should be directly evaluated in future clinical trials. The study was not powered to detect changes in cognition, however ADAS-Cog measurements tended to worsen in the placebo group while there was no change in the MSDC-0160-treated group (Shah et al., 2014).

There has been more detailed study of the mechanistic action of MSDC-0160 in pre-clinical models of PD. Ghosh et al. (2016) demonstrated that MSDC-0160 could directly protect against 1-methyl-4-phenylpyridinium (MPP+) insult in murine and cultured human midbrain dopamine neurons. This effect was caried over to mice that were treated with methyl-4-phenyl-1,2,3,6-tetrahydropyridine (MPTP)– where MSDC-0160 treatment improved locomotor behavior, increased survival of nigral dopaminergic neurons, boosted striatal dopamine levels, and reduced neuroinflammation. Similar results were obtained in the slowly progressive Engrailed1 (En1^+/−^) genetic mouse model of PD that demonstrates a progressive loss of the differentiated status of dopamine neurons. Progression of neurodegeneration in all of these cellular and mouse models of PD was accompanied by both an increase in mTOR activation and increased inflammation. In each case, the beneficial effects of MSDC-0160 treatment coincided with a reduction in mTOR activity and a decrease in inflammation toward the levels that occurred in the control models. MSDC-0160 treatment also prevented dopamine neuron loss in both an MPP+ and α-synuclein–based *Caenorhabditis elegans* model. Selective knockdown of either the C. *elegans* orthologs of MPC1 or mTOR prevented the protective effects in these worms. MSDC-0160 treatment was not effective in rodent models of alpha-synuclein overexpression that did not exhibit underlying inflammation or defective autophagy (Peelaerts et al., 2020).

Quansah et al. (2018) summarized the direct effects of MSDC-0160 to attenuate pathologically-activated mTOR and inflammatory pathways associated with regulating autophagy in both neuronal and glial cells. In glial cells, treatment with MSDC-0160 also attenuated the inflammatory response to the bacterial toxin LPS. Interestingly, the treatment with MSDC-0160 protected mitochondria from the loss of oxygen consumption that otherwise occurred both in neuronal cells exposed to MPP + toxin or a glial cell line exposed to LPS. Thus, the evidence suggests that preservation of mitochondrial function in the face of these challenges may be related to the downstream neuroprotective and anti-inflammatory action.

In further support of the importance of pyruvate metabolism in affecting PD pathology, Mallet et al. (2022) have recently demonstrated that treatment with MSDC-0160 treatment improved motor behavior, decreased dopaminergic denervation, and reduced mTOR activity and neuroinflammation in the unilateral 6-OHDA rat model of PD. Metabolomic analysis indicated that reprogramming pyruvate metabolism increased ketogenesis, beta oxidation and glutamate oxidation and that multiple downstream pathways were likely involved in the beneficial effects. Thus, while many molecular and metabolic mechanistic details remain to be defined, modification of pyruvate metabolism at the level of the MPC with compounds such as TZDs can be neuroprotective.

## Metabolic Connection to Molecular Mechanisms in Common to AD and PD

**FIGURE 1:**
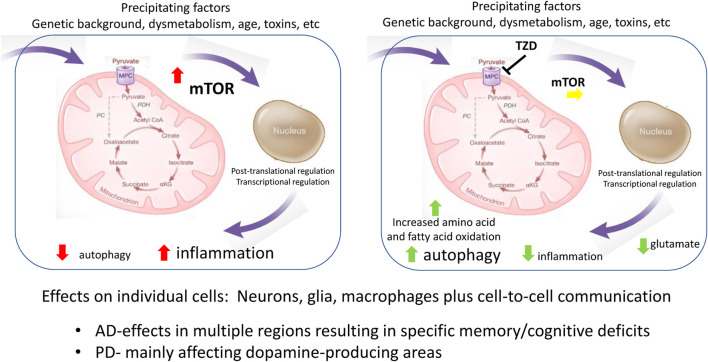
Metabolic connections to molecular mechanisms common in AD and PD.

The collection of data summarized here point to the importance of metabolism in the progression of both AD and PD. While the milieu including glycemic state is likely important (Cardoso and Moreira, 2020; Cheong et al., 2020), we have summarized how underlying features relating to cell metabolism, particularly as related to mitochondrial function in specific cells, connect molecular mechanisms contributing to the pathology. For example, the direct effects of mitochondrial-directed TZDs on neurons and glia mitigate against reduced autophagy and increased inflammation. Growing evidence is connecting the MPC, now known to be a direct molecular target of the first generation TZD insulin sensitizers, to function in multiple cell types. Figure 1 summarizes some of the currently recognized sites of interaction that map onto cell processes involved in disease progression. The immediately recognizable points of influence are autophagy and inflammation, which have been shown to be important in both glia and neurons, but which are obviously important to regulation in many cell types. The multiple molecular processes involved in this regulation include nutrient sensing processes such as mTOR and downstream regulatory transcriptional networks regulated both by metabolites, redox, and oxygen levels which can modify post-translational and epigenetic processes (Bertogliat et al., 2020). The physical regions where the neurodegeneration occurs define the specific syndrome. For example, failure to clear misfolded proteins related to dopamine-producing neurons will produce PD-like disease, while reduced function in memory and executive function centers will define a different set of symptoms.

**FIGURE 2:**
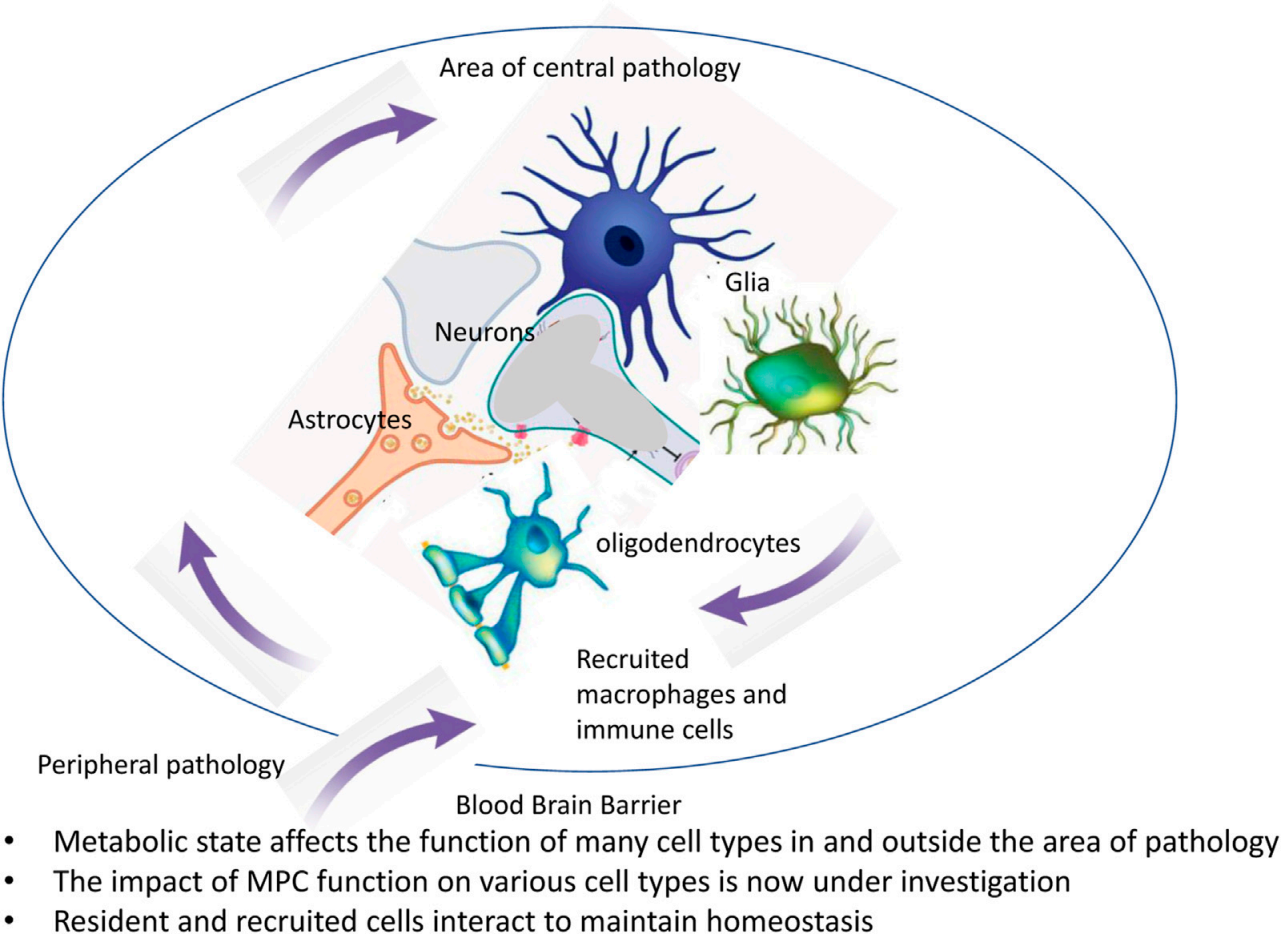
Involvement of multiple cell types in pathology and in repair mechanisms.

Outcomes of disease progression and mitigation involve effects on multiple cell types and also might involve intercellular communication between various cell types of the brain. This has important implications for a range of processes from maintenance of synaptic connections to regulation of cerebral blood flow and involves cell types not routinely considered in drug development programs aimed at specific neurodegenerative diseases (Figure 2). Ongoing work is defining the specific aspects involved in individual cell types using cell-selective knock out of the MPC. This work will help define which processes in which cells have the most important impact of specific aspects of AD or PD. It is also important to note that whereas the brain is usually considered privileged with respect to immune regulation because of the blood brain barrier, there are changes to blood brain barrier with aging and dysmetabolism which can also contribute to inflammation, neurodegenerative processes, and dementia (Nortley et al., 2019; Guo et al., 2022; Kurz et al., 2022). A more comprehensive understanding of interactions between cells and their regulation by the metabolic milieu/changes in mitochondrial function might also benefit from the study of more complex systems *in vitro* (Cenini et al., 2021).

In conclusion, there are multiple molecular mechanisms associated with metabolism that impact the progression of AD and PD. This is likely true of age-related neurodegenerative issues in general, some of which may fall outside narrow clinical definitions. The identification of the MPC as a target of the insulin sensitizer TZDs provides a logical explanation for the pleotropic effects of these compounds. The elucidation of these common pathways that mediate disease progression may facilitate new approaches to disease-modifying treatments for AD and PD.
